# Address on Dental and Oral Surgery

**Published:** 1886-08

**Authors:** John S. Marshall

**Affiliations:** Chicago, Ill.


### Address on Dental and Oral Surgery.

(Delivered at the Thirty-seventh Annual Meeting of the American Medical Association.)

BY JOHN S. MARSHALL, M.D., OF CHICAGO, ILL.

One of the duties of a Chairman of a Section is to examine, sift, and weigh the material that has accumulated during the year in relation to the department of medicine over which he may preside in this Association, and which may have more or less legitimate claims upon the profession as new discoveries, new theories, or improvements in methods of operating, or treatment, and to bring to your notice such of them as are in his judgment of greatest importance. This I have found to be by no means an easy task. My report, however, will be brief.

### TRANSPLANTATION OF TEETH.

Let me first call your attention to a matter which has somewhat startled the scientific world, and particularly that portion of it interested in dental and oral surgery, viz.: the discovery made by W. J. Younger, M.D., of San Francisco, California, that teeth can be transplanted into artificial sockets drilled in the jaws, and apparently made to unite with the surrounding tissues as firmly as though placed there by the natural process of dentition.

The operation of transplanting teeth from the mouth of one individual into the natural alveoli of another was first performed by John Hunter, but it soon fell into disrepute from the annoyances and dangers which so often followed it, among which may be mentioned immediate suppurative inflammation, which prevented the union of the tooth with the alveolus; subsequent alveolar abscess, the result of the putrefaction of the pulp; necrosis of the jaw, and occasionally tetanus.

The revival of this operation, and the introduction of *replanting* teeth as a curative measure for alveolar abscess, under antiseptic precautions, a decade or more ago, viz.: the extirpation of the pulp, the filling of the pulp canal, and the washing of the tooth and alveolus with some one of the antiseptic preparations, has placed it among the successful operations, so far as the immediate annoyances and dangers before mentioned are concerned. But the operation really cannot be called successful even in those cases where firm union has taken place, for in very many of them, sooner or later, resorption of the root occurs similar to, if not identical with, that phenomenon which takes place in the process of shedding the deciduous teeth, and the tooth becomes loose, and either drops out or has to be extracted. The failures in the operation of transplanting have been variously estimated at from 25 to 50 per cent., but when we take into consideration the fact that very many of the teeth thus treated fail after a year or two of union, the percentage must be very much higher.

Quoting from the *Pacific Medical and Surgical Journal and Western Lancet*, January, 1886, Dr. Younger says: "*But transplantation can be made a success and void of all danger and unpleasant consequences, if only common sense, cleanliness, and ordidary skill and care are taken.*" And it is to prove this and unprejudice the scientific mind, and through it the public, and do away with the abomination of false teeth, that he "presents to the profession the results of his experiments and experience in this direction.

"He gained courage to try the operation by reflecting on the experiment of John Hunter, who to test the vitality of the pericementum, planted a tooth in a cock's comb. This tooth attached itself firmly to the crest, and a few months afterwards the cock was killed and microscopical examination showed that a living union had taken place, the blood-vessels of the comb and pericementum having established free communications."

Dr. Younger "also tried the experiment upon a cock's comb, to further and personally assure himself of the truth of this statement, and confirmed, as far as attachment was concerned, the experiment of the great surgeon. But in his experiment, he took the precaution of removing the pulp and filling the pulp chamber and root canal with a preparation of gutta percha much used by dentists for temporary fillings. This was in order to avoid any trouble from a decomposing pulp. The tooth was then well cleansed with warm water and dipped in a disinfecting solution.

"The success of this experiment satisfied him that the pericementum would attach itself to any vascular body, and that if properly planted in a fresh socket it would attach itself and form a living union with the surrounding tissues, without the production of after-pains or other evil consequences."

The first practical experiment upon the human subject of transplanting a tooth into a natural alveolus performed by Dr. Younger was on January 24, 1881. The tooth was treated in the same manner as that described for transplantation in the comb of the cock: the alveolus was washed out with a disinfecting solution, the apex of the root trimmed to make it of the desired length, and then pressed into position, and retained by delicate silk ligatures attached to adjoining teeth. At the end of four weeks the ligatures were removed and the tooth found to be firmly attached to the surrounding walls of the alveolus, and at the time of writing his paper, was still doing good service.

Dr. Younger reports "between thirty and forty cases of transplanting into sockets already formed"—natural sockets—with "but two failures;" one due to the neglect of the patient, and one to his own inexperience. These are remarkably good results, so far as they go, but a sufficient length of time has not yet elapsed to prove them permanently successful, for resorption of the roots may yet take place and the teeth be lost. This has been the experience of many of the most careful and painstaking operators, and I shall be greatly surprised if in some of Dr. Younger's cases which now appear to be successful, this does not prove to be the final outcome.

Thus far in the experiments of Dr. Yonnger, he has traveled over the same ground previously traversed by many others of his professional brethren, but from this point he enters a new and entirely unexplored field, and first introduces the novelty of making a garden or nursery of a cock's comb in which he plants freshly extracted teeth, and thus retains their pericemental vitality for an indefinite period, or until such time as a favorable opportunity presents to transplant them into the human mouth. Let me quote him upon this point. He says: "The great and only difficulty I had to contend with was the procurement of teeth at the time they were needed. At last a way suggested itself. I applied to my dental friends for whatever good teeth or roots the exigencies of cases required them to extract. The experiment of Hunter and my own experience had taught me that teeth could be kept alive, indefinitely, in cocks' combs. But could they be transplanted to the human mouth again and made to grow there? I concluded they could, and my first experiment verified my conclusion. On November 28, 1885, a bicuspid that had been in a cock's comb for ten days was transferred to the mouth of a gentleman, where it had fastened itself, as if there had been no gallinaceous period in its existence.

"Where he has not been able to procure a suitable tooth, he has taken a root and mounted an artificial crown upon it. Sometimes he used the natural crown of a tooth that had been irretrievably loosened by incrustations of tartar on its roots. In this case he simply saws off the bad root and attaches a good one to its crown by means of one or more gold screws and cement. In these cases the patient simply changes roots. He says: I have also discovered that the pericementum can be kept alive for certainly two days in warm water, temperature 100° to 110° F. He has in two cases transplanted teeth successfully that had been so kept for fifty hours."

He next describes how he was led to the discovery of the fact that teeth can be transplanted into *artificial alveoli* drilled in the bones of the jaws, and made to unite with the surrounding bony tissues and the gum. "His former practice, when he found a root was too long or too wide for the socket, was to cut off from the apical extremity, or shave off from the surface of the root, the necessary quantity to insure a fit; but so often the best portion of the pericementum was in that way removed, that he tried deepening or widening the cavity as the case required, often cutting freely into the bone in order to save all possible of this valuable pericemental tissue. And he found that adhesion took place in this portion as perfectly as in the unbroached. The consideration of this led him to the grand conclusion that artificial sockets could be drilled into bone itself and teeth planted therein as successfully as into the natural cavities."

The first operation of this kind was performed upon a young lady on June 17, 1885, and up to the month of January of the present year he reports having made the operation *seven* times in all. "Four teeth were transplanted in the mouth of one patient, and one each in three others, the original teeth having been lost for periods varying from two to twenty years. All of these are reported as entirely successful, no serious after-trouble following the operation, and the union is perfect with the jaw and the gum."

The first operation, which is a fair sample of all the others, is described as follows: "Miss W., a young lady of twenty-four years, had lost the left superior lateral incisor, root and all, four years previously, and had been wearing as a substitute an artificial tooth on a rubber plate. The collapse of the gum, consequent on the absorption of the alveolus, was so great and the exposure of (artificial) gum so much, in conversation, and especially in smiling, that the falsity of the denture was immediately recognized, and was an object of great distress to her. As it was impossible, for the reasons just given, to produce an artificial substitute that would look natural, he determined upon the following operation: Taking a corresponding lateral from the mouth of a young man, which, from its awkward position, was disfiguring his appearance, he prepared it as he does all teeth used in transplantation, viz.: removed the pulp, filled the pulp chamber and root canal with Hill's stopping, and finished the apex with gold. The tooth was then placed in water of the temperature of 100° to 110° F., to cleanse it of all blood and impurities, and allowed to remain about one hour. It was then placed in a bath of bichloride of mercury, 2 parts to 1,000 of water, for about fifteen minutes, to disinfect it. The tooth being now ready, attention was turned to the patient. A hole was cut in the gum a little less than the diameter of the root to be inserted. He then took an ordinary flat, angular-edged drill, and drilled into the bone in the line of direction the tooth was to occupy. When fully deep enough, the cavity was widened and the socket formed with a cone-shaped burr. When it was found by trial that the cavity would receive the tooth perfectly, it was carefully washed and sponged, in order to remove every particle of detached bone, first with warm water, then with cold, and lastly with the bichloride solution already referred to, and when the bleeding had ceased, the tooth was introduced and kept in position by delicate silk ligatures attached to the central incisor on the right and the canine on the left. There resulted a little swelling over the root, which remained a few days and then gradually disappeared. An accident occurred to the gum during the development of its socket. Just as the drill touched the surface of the bone, the young lady suddenly moved her head, which caused the instrument to slip forward and through the gum, making a triangular-shaped gash of fully an eighth of an inch in length. Before the tooth was inserted, the edges of this wound were brought carefully together and retained in contact by delicate silk sutures. On the fourth day the sutures were removed and no mark was apparent to tell of the lesion that had existed. In twelve days the ligatures were removed from the tooth and it was found to be well attached. About three weeks afterwards, the gum being free from any sign of irritation and the tooth comparatively firm, and as it was desirable to improve the position of the right superior central and lateral, ligatures were passed around the new tooth. This, unfortunately, set up a slight inflammatory action, and an abscess formed a few days after, and a little discharge of pus took place. The ligatures were immediately removed and the abscess treated with injections of iodine. And he says: When last seen the abscess had entirely disappeared, the surrounding gum had resumed its normal appearance, the tooth became firm in its position, and is now performing its functions in common with its fellows as though it had never been a stranger in the mouth." Dr. Younger publishes the names of several medical gentlemen, some of them members of this Association, I believe, and also the names of several dental practitioners known to the profession, who have examined these cases in a critical manner and have expressed themselves as "fully satisfied with the success and utility of this operation."

In an addendum to this paper Dr. Younger offers an explana-nation of the process of union, which takes place in this class of cases between the tooth and the bone into which it is placed. These views may be briefly summarized as follows: The dental alveolus has no periosteal lining, and the pericemental membrane which covers the root of the tooth—be it a single or double membrane—"has a callus generative energy" only upon that side in contact with the tooth, while the outer surface "has simply the power of forming attachment." To the *endosteum* which lines the cells and interstices to the surrounding bony tissue, he believes, belongs the power of forming the alveolus about the tooth, whether the tooth is erupted by the physiological process of dentition, or artificially implanted.

To prove this theory he proposes to commence (for publication) a series of experiments upon rabbits, by drilling into various parts of their osseous tissue, and implanting roots of teeth into these cavities, and then at various intervals to kill the rabbits and make critical microscopical examinations of the existing conditions of the implanted roots and the surrounding bony structure.

Of the immediate success of Dr. Younger's operations there cannot be a shadow of doubt, for the evidence is supported by unimpeachable witnesses. But the permanent success of the operation is not yet assured. It will require a much larger number of operations to establish its practicability, and several years to prove it permanently successful. It is to be feared that the same causes which have operated against the permanent stability and usefulness of so many teeth replanted and transplanted into *natural* alveoli, would operate against those teeth transplanted into artificial sockets, and would therefore advise that we "make haste slowly" in adopting so radical and startling an operation; but I trust these fears may prove entirely unfounded, and that the introduction of this operation may, as Dr. Younger hopes, "do away with the necessity for false teeth, and so add immeasurably to the comfort, beauty, and health of the human mouth."

Next let me call your attention to the operation of

### SPONGE-GRAFTING IN THE MOUTH.

This subject of sponge-grafting has for the last two or three years been receiving considerable attention by way of experimentation from specialists in dental and oral surgery; but operations in the direction of restoring lost tissue in the mouth by this means, have until lately met with very limited success. During the last year much better results have been obtained, consequent largely upon the improved methods in preparing the sponge, in protecting it against septic influences after being placed in position, and in lessening its liability to become displaced. Much greater difficulties have to be overcome in using the sponge-graft in the mouth than upon the external surfaces of the body. In the latter the usual antiseptic dressings are all that are needed to protect the sponge and wound from the entrance of the micro-organisms, and to retain it in position; while in the former no such dressings can be employed, as they immediately become saturated with the oral secretions and fouled by the introduction of food into the mouth. The sponge also needs to be prepared in such a manner as to render it as nearly *permanently* antiseptic as possible, for the reasons just mentioned.

The method generally followed in preparing the sponge for grafting is that introduced by Dr. Edward Borck, of St. Louis, viz.: to remove all the earthy matter that might be contained in the sponge by placing it in dilute hydrochloric acid, and then, after washing it, to render it antiseptic by treating it in iodoform dissolved in sulph. ether, the sponges afterwards to be dried and excluded from the air by being placed in tightly corked bottles. Sponges, however, prepared in this manner, if kept for any length of time become shriveled and soft, and soon disintegrated, thus rendering them useless, and making it necessary to prepare a fresh sponge for each case.

To Dr. Wm. H. Atkinson* of New York, belongs the credit of suggesting an improvement in the preparation of the sponges and in the means of retaining the graft in position. He says "choose fine surgeon's sponges, free them from foreign elements," and then place in a "sterilizing solution made by adding one grain of bi-chloride of mercury to one oz. of distilled water," and then raise the temperature to 130° F. and maintain it at that degree for from ten to thirty minutes. He adds a word of caution in regard to the heating of the sponges, for if the temperature is allowed to go much above 130° the sponge is likely to be spoiled, for albumen begins to coagulate at 133°, and is cooked if it gets beyond 163° to 164°, aud is then "not fit to be wrought into tissue." Sponges treated by this method can be kept for an indefinite period if placed in the above sterilizing fluid and excluded from the atmosphere.

The methods which he suggests for protecting sponge-grafts in the mouth from the dangers of being displaced by mastication or in cleansing the teeth, etc., is to form a splint from thin platinum plate, which shall cover the parts and be closely adapted, and at the same time free from pressure over the graft.

The class of operations in which Dr. Atkinson has been most successful are closing the pus-pockets resulting from pyorrhea alveolaris, the reproduction of lost alveolar and gum tissue, and the healing of chronic alveolar abscesses.

Few operations in the mouth outside of these just mentioned have yet been attempted, but there is every reason to believe that with care and skill, perforations of the hard and soft palates, the result of surgical operations, injuries, or specific disease may

*"Transactions American Dental Association," 1885, p. 149.

be successfully closed by it, and lost parts in other locations of the mouth and face, more or less completely restored by the same means.

The success of the operation, however, when made in the mouth will depend very largely upon our ability to prevent the contamination of the sponge with septic organisms This in most cases may be accomplished by frequently and freely washing the parts with peroxide of hydrogen, followed by the bi-chloride solution, 1 in 1000.

Finally, allow me to direct your attention to the

OPERATION FOR HARE-LIP AND CLEFT PALATE

in early infancy, as performed and strongly advocated by Dr. D. H. Goodwillie, of New York. In these cases there has been a failure of union during inter-uterine development between the maxillæ and the lateral halves of the lip, and a cleft is the result. In this condition the anatomical relations of the parts are changed, both in form and dimensions. The maxillæ now separated cause an abnormal increase in their dimensions, and when extended forward producing a single or double hare-lip; there is also great distortion of the face.

Dr. Goodwillie's method is to replace the separated and distorted maxillæ and nose to their normal relations by *manual force* repeatedly applied. But in order to accomplish this with the least difficulty, it must be done as soon after birth as the conditions and circumstances surrounding the child will permit, and before ossification has had time to advance. When the bony frame-work has been restored to its anatomical position, then the operation on the cleft lip and soft palate should immediately follow. For the operation of staphylorraphy and uranoplasty the child is etherized and confined in a metallic jacket and head rest. The patient is then placed in a good light, and in such a position that the surgeon standing with the head of the patient against his breast, can, by looking over, command a full view of all parts of the palate. The arms of the operator rest upon the head of the patient and thus enable him to control his movements. The oral speculum is then placed in position and the assistant holds down the tongue by means of a hollow tongue-holder and etherizer combined, through which anæsthesia is maintained during the operation. This is a very great advantage, as it saves valuable time, which is otherwise lost by frequent halts in the operation to reapply the anæsthetic.

The divided uvula is then seized with the forceps, and with the small pointed knife the edges of the cleft are pared from the uvula forward to the anterior point of the cleft. The ligatures are then passed, beginning at the posterior part of the cleft, by means of a hollow needle and holder, armed with silk-worm gut; the point of the ligature is then pushed through the needle and grasped by the forceps and the needle withdrawn. The ends of the ligature are secured temporarily by means of small leaden clamps; this is repeated until the required number are passed. The ligatures are afterwards tied with the fingers, commencing, as a rule, with the posterior one.

If any point appears to be unduly strained by the ligature, a suture-pin clamp is adjusted to relieve the tension upon the sutures and is allowed to remain until union has taken place. The methods of dividing the levator palati and palato-pharyngeal muscles are those in common practice.

In uranoplasty, the soft tissue covering the hard palate is either lifted from the bone by means of periosteal elevators, or lateral incisions are made through the soft tissue and the bone drilled and split, and afterwards brought together and held in position by ligatures and clamps, which are permitted to remain for from six to twelve days.

To prevent the child from getting the hands to the mouth, leather elbow-pads are strapped upon the arms.

The instruments used for these operations by Dr. Goodwillie are of his own designing, and meet a long-felt want on the part of oral surgeons, and convert an otherwise tedious and disagreeable operation into one of comparatively slight inconvenience. —*Jour. Am. Med. Assn.*

